# Children and youth in the climate crisis

**DOI:** 10.1192/bjb.2021.16

**Published:** 2021-08

**Authors:** Ann Sanson, Marco Bellemo

**Affiliations:** 1 Department of Paediatrics, University of Melbourne, Australia; 2 School Strike For Climate, Australia

**Keywords:** Post-traumatic stress disorder, anxiety disorders, climate change, childhood experience, developmental disorders

## Abstract

This editorial is co-written by a developmental psychologist and a young climate activist. We start by showing how the climate crisis is imposing a heavy psychological burden on children and youth, both from experiencing climate-related disasters and from the knowledge that worse is to come. We then describe the global movement of youth demanding urgent climate action. We conclude that health professionals can support young people in many ways, but particularly by supporting their capacity to take action, raising awareness about the impact of the climate crisis on youth mental and physical health, and taking action themselves to work for a secure climate future.

## The climate crisis poses an existential threat

Climate change is well underway and poses a critical threat to the future. Although the Earth is currently only about 1.1°C above preindustrial levels,^1^ it is clearly already too hot.

The Global South (also referred to as low- and middle-income countries or the majority world) is currently bearing the brunt of climate change effects, but they are rapidly becoming more common globally; for example, unprecedented wildfires in the Amazon, Australia and California, record-breaking hurricanes in North America and record-breaking heatwaves in the UK and Europe. Glaciers and icecaps are melting faster than predicted, rising sea levels have already forced coastal and island populations to relocate, and droughts and resource shortages have already contributed to conflict and refugee crises.^2^ Global warming is contributing to the Earth's sixth mass extinction, with about 1 million animal and plant species under threat.^3^ All these serve to heighten young people's awareness of the reality of the climate crisis.

We are fast approaching irreversible tipping points and, without moving speedily to net zero emissions and draw down the excess greenhouse gases already in the atmosphere, we face an existential threat to the global ecosystem and human civilisation.^4^ The well-being and even survival of today's children are at risk. As Aran Cosentino (Italy, aged 18 years) said, ‘I am worried about climate change because the future of us young people is in danger. If governments do not act quickly, we will not have a future where we can live in peace with our children’.

## Impact of climate change on child and youth physical and mental health

Both the causes and consequences of climate change are inequitably distributed. It has been disproportionately caused by the Global North, also referred to as high-income countries, the minority world or the WEIRD nations (Western, educated, industrialised, rich democracies), comprising 12% of the world's population,^5^ However, it will most severely affect the Global South,^6^ where 85% of children live^7^ but the institutional capacity to respond is weaker.^8,9^ Further, although predominantly caused by the current generation of adults, the worst consequences will occur during the lifetimes of today's children and young people,^10^ as reflected by Emma Demarchi (Australia, aged 17 years): ‘Young people are facing climate related inter-generational theft and its effects on us are astounding’. Also, climate change will also reinforce societal inequalities based on income, ethnicity, race and class. For example, the United Nations International Children's Emergency Fund (UNICEF)^11^ vividly illustrates how climate change exacerbates pre-existing socioeconomic inequalities. When confronted with an extreme weather event, like a drought, a child from a wealthier family may experience temporary hardship, but can recover relatively fast – their family can safely relocate and stay together, purchase food even when prices escalate, access clean water even when scarce, access healthcare even when services are overstretched, and maintain the child's education and reduce other social stressors. None of these may apply for a child in poorer circumstances, who may find themselves on an escalating downward pathway through the accumulation of negative repercussions, to result in long-term, and sometimes lifelong, deprivation.

Climate change has both direct and indirect effects on the physical and mental health of children and youth. Because of their immature physiological systems, reliance on adults and likely repeated exposure to climate events over time, children are more vulnerable to the negative effects of climate change than adults.^12,13^ Both sudden extreme weather events and slower, longer-lasting climate effects (e.g. rising sea levels, droughts) are increasing in frequency and ferocity. These, in turn, directly cause deaths and injuries, physical ill health (e.g. through malnutrition, lack of clean water, infectious diseases) and mental health problems, including post-traumatic stress disorder, anxiety and depression, phobias and panic, sleep disorders, cognitive deficits and intellectual disabilities.^14–16^ As one example, after floods in Pakistan, 73% of 10- to 19-year-olds displayed high levels of post-traumatic stress disorder symptoms, with displaced girls affected most seriously.^16^ Reactions to climate disasters also include distress, grief, anger, feelings of helplessness and hopelessness, and increased aggression and violence.^17^

More broadly, climate change threatens the underlying social, economic and environmental determinants of children's health.^18^ By disrupting families and communities, climate change can have an indirect impact on children; for example by increases in rates of domestic violence and child abuse after hurricanes and wildfires.^19^ Climate change causes and exacerbates conflict^20^, being described as a ‘threat multiplier’ for intra- and interstate conflict.^21^ There are already millions of so-called ‘climate refugees’, forced to seek new homes, where they are often met with hostility and resistance.^22^ The resultant disrupted parenting, education and social connections all have long-term sequelae for children and youth.^16,23^ Particularly for Indigenous youth, breaking their connection with the land can lead to mental health problems, including depression, despair and suicide.^24^

## Emotional responses to the climate crisis

Although adults may wish to protect children from knowledge of the climate crisis, this is neither possible nor right: the United Nations Convention on the Rights of the Child^25^ confirms their right to know about, and be involved in, issues that will affect their future. Research around the world shows that most children and youth know about the climate crisis and are worried about it. A 2013 survey of British 11- to 16-year-olds found that 74% were worried about its impact on their future.^26^ A more recent study of Australians aged 7–24 years indicated that 96% considered climate change a serious problem, and 89% were worried about its effects.^27^ Even more recently, 16 young people summarised the results of a survey of 1477 young Australians (aged 10–24 years) as thus: ‘We are aware of climate change, and we are worried about climate change. We are concerned about the repercussions of climate change globally, regionally and here at home. We are concerned about experiencing climate-related disasters due to a lack of action by those in power, and we are worried about what that means for us, our families, and our friends, in the present, and in the future’.^28^ A recent survey by the Royal College of Psychiatrists^29^ found that 57% of child and adolescent psychiatrists reported seeing children and young people distressed about the climate crisis and the state of the environment.

The emotional impact of knowing about the climate crisis is not limited to ‘worry’. Children and youth also experience anger, frustration, depression, sadness, grief, anxiety and a sense of powerlessness about its impact on their lives.^26,30^ Interviews with 10–12-year-olds in the USA found that strong feelings of fear, sadness and anger were expressed by 82% of the children.^31^ In a 2019 survey of over 15 000 Australians aged 14–23 years, one in five reported planning to have no children, or fewer children, because of their concerns about climate change,^32^ as illustrated by Alice Hardinge (Australia, aged 23 years): ‘My future feels dire, and my decision to have children has been impacted by the fear of food shortages, droughts, fires and floods’.

Burgeoning rates of ecoanxiety (severe and debilitating worry) and ecological grief (grief related to current or anticipated ecological loss) are commonly described.^33^ However, despite these emotional reactions being uncomfortable and, in extreme cases, debilitating, it should be recognised that they are based on a rational understanding of the climate science, and hence should not be regarded as unreasonable, illogical or pathological.

## Children and youth taking action

For decades, children and youth have been responding to their knowledge of, and fears about, the climate crisis by demanding climate action from older generations and those in power. In the past 2 years, Greta Thunberg has been the face for the millions who have mobilised across the world, but back in 1992, 12-year-old Severn Cullis-Suzuki gave a speech at the United Nations Rio de Janeiro Earth Summit that ‘silenced the world for 5 minutes’. Severn said to world leaders, ‘You grown-ups say you love us. But I challenge you, please, make your actions reflect your words’.^34^ Almost three decades later, in 2018, Greta Thunberg made a very similar speech to world leaders at the 24th United Nations Framework Convention on Climate Change (COP24) in Katowice, Poland: ‘You say you love your children above all else, and yet you are stealing their future in front of their very eyes’.^35^ The similarities between the two speeches, almost three decades apart, starkly show how little has changed and how children and young people are right to feel frustrated.

Greta Thunberg began striking from school on 20 August 2018, vowing to stay until the Swedish election 3 weeks later. Initially it was just Greta and her sign ‘Skolstrejk för klimatet’ (‘school strike for the climate’), but from the second day others started joining her. After this first strike, Greta began boycotting class every Friday, announcing the strikes as ‘Fridays For Future’, which quickly gained international attention.

Showing how her actions resonated with children around the world, just a few months after Greta's first day of striking, school students across the world had coordinated national school strikes involving thousands of children and youth, such as in Australia, where over 150 000 students took action in November 2018. The first global day of action followed on 15 March 2019, with strikes in over 100 countries, and by 20 September, just 13 months from the first climate strike, an estimated 4–6 million people participated in >2500 events in over 163 countries. This is estimated to be the largest climate mobilisation in world history.^36^

Despite these massive displays of child and youth fear, anger, frustration and determination, their demands have ‘not translated into action’ and emissions have continued to rise whilst ‘the changes required are still nowhere in sight’, as Greta Thunberg said to world leaders at 25th United Nations Framework Convention on Climate Change (COP25) in Madrid, Spain, on 11 December 2019.^37^ Now, in a time of global unrest, with concurrent crises facing young people, they continue to organise mobilisations with demands centred in an understanding of the fundamental ways in which social and economic issues are interlinked with the climate catastrophe.

## Action is the antidote to despair

Conversations with students who have engaged in school strikes or other forms of activism show how their activism has helped them manage their anxiety about the future and channel it into determination, courage and optimism. As Alice Hardinge said, ‘Climate despair is real and dangerous, the best cure is action … [taking action] creates a sense of solidarity, of cooperation and productivity in the face of despair’. This idea that ‘the best antidote to anxiety and despair is action’ suggests that an important way to build young people's resilience, self-efficacy and agency is by encouraging and supporting their involvement in activities to both mitigate and adapt to climate change.^38^

Young people involved in climate activism appear to have learned many valuable positive skills and attributes through their involvement. The Melbourne School Strike organiser and volunteer for the Australian Youth Climate Coalition, Andeli Zuz (Australia, aged 20 years), said: ‘Without the skills I learned in activism I simply would not have been able to do this job, as simple as that. It has taught me far more about community organising and event management than school ever could’. Another Melbourne school strike organizer, Emma Demarchi said: ‘Taking action on climate change can be incredibly rewarding and fulfilling and has certainly built many of my skills up’.

It is interesting to note that these skills and capacities which young people report developing through taking action on the climate crisis match well with those describing positive youth and young adult development,^39,40^ including self-regulation of behaviour and emotions, ‘bigger-than-self’ values such as social justice, conflict resolution skills, teamwork skills and social and civic engagement skills. As Alice Hardinge said: ‘I've learnt how to …  talk to authority figures, recognise my rights, speak confidently in public …  not let keyboard warriors impact my self-worth … communicate non-violently and how to work effectively in a non-hierarchical and consensus based collective’. These skills will stand them in good stead throughout their lives.

Yet, although taking climate action is highly rewarding and beneficial for young people, the burden and scale of the climate crisis is often overwhelming. For example, Emma Demarchi said: ‘Climate action can also very often feel just as lonely and full of despair and anxiety. Young people often feel like they are fighting a battle they need to win but know they might never and the impacts on mental health can be great’. Andeli Zuz commented: ‘Sometimes it [climate action/advocacy] makes me feel empowered, like I have some control, other times deflated as I feel like no matter what I do it won't work’.

Young people who feel immense pressure and responsibility to do all they can to protect the future are thus at risk of burnout and mental strain. To sustain their mental health, engagement and empowerment, strong communities and support networks that are honest and hold space for their feelings are vital. Mental health professionals have an important role in validating such feelings, helping young people manage them and supporting their activism. It is encouraging to see some resources to support such work now being developed. For example, the Royal College of Psychiatrists has recently produced a resource to help young people cope with ecodistress.^41^ However, equally critical is to demonstrate to young people that they are not being asked to take the whole burden themselves; this entails mental health professionals themselves taking action, and using their influence and expertise to work for speedy and effective policy change to help secure a habitable planet and a safe future for the next generation.

There are other important roles for mental health professionals. For example, in response to the confusion that many parents express about how to talk to, and support, their children in the context of the climate crisis, parent-focused resources were developed by the Australian Psychological Society,^42,43^ and can be used in community workshops. Not all children and young people, nor all mental health professionals, are aware of the implications of the climate crisis for the next generation, creating an ongoing need for education in work places, practice and through written material. It also needs to be acknowledged that facing up to the reality of the climate crisis is challenging for mental health professionals themselves. In Australia, Psychology for a Safe Climate (https://www.psychologyforasafeclimate.org) has developed resources and methodologies for supporting activists, including health professionals, to manage their climate grief. In advocating for policy change, strong position statements from our professional organisations can be a valuable tool.

## Concluding comments

The climate crisis is already placing significant psychological burdens on children and young people, from both direct experience and simply knowing the dangers it poses for their future. Yet until recently, children's voices have been neglected in discussions of the climate crisis. But the courage and determination of Greta Thunberg acted as a catalyst for children in their millions to raise their voices and demand to be taken seriously, and to demand action.

Mental health professionals can help to protect the next generation and prepare them for the future. Clinicians need to be aware of how the climate crisis can cause emotional distress, and recognise and respond to the psychological consequences of exposure to the effects of climate change, especially in the Global South, where psychological help is scant.^8^ Supporting young people in speaking out and taking action, whether to protect their communities from the effects of climate change or to demand action by politicians and others, may be the most beneficial approach that mental health professionals can take. Such action builds beliefs in self-efficacy and collective efficacy, practical active citizenship skills, courage and hope,^9,13^ which is reinforced when young people can see that mental health professionals are also taking action. This editorial seeks to help give voice to youth and provide an example of intergenerational partnership.

However, without speedy action at a global scale to prevent catastrophic climate change, it will not be possible to protect young people's psychological well-being, or even their survival. Today's adults may be the last generation that can ensure a liveable world for future generations. They need to act as citizens to demand effective and speedy climate action, and not rely on young people to carry this burden alone. For mental health professionals who have responsibility for protecting human health and well-being, there is a particular moral imperative to use their status and expertise, individually and collectively, to speak out on behalf of the children and youth of today and tomorrow.

## Ethics and consent

No ethical approval was required for this editorial. Young people provided quotes voluntarily and explicitly agreed to them being used. This paper includes quotes volunteered by young people aged 17–24 years, who were known to the second author through their joint engagement in the school strike movement. They were invited to provide responses to a series of questions for the purposes of this editorial, and gave their written consent to them being used herein.

## Data Availability

The quotes used in this editorial were provided specifically for this purpose. Access to them would require further consent from the participants. The corresponding author (A.S.) can be contacted for further information.

## About the authors

**Ann Sanson** (PhD) is an Honorary Professorial Fellow at the Department of Paediatrics, University of Melbourne, Australia. **Marco Bellemo** is an organiser with School Strike For Climate in Australia.

## References

[1] World Meteorological Organization (WMO). WMO Statement on the State of the Global Climate in 2019. WMO, 2020 (https://library.wmo.int/index.php?lvl=notice_display&id=21700#.X9xBdC2r0dU).

[2] United Nations High Commissioner for Refugees (UNHCR). *Climate Change and Disaster Placement.* UNHCR, no date (https://www.unhcr.org/en-au/climate-change-and-disasters.html?query=climate%20refugees).

[3] Intergovernmental Science-Policy Platform on Biodiversity and Ecosystem Services (IPBES). *Nature's Dangerous Decline ‘Unprecedented’; Species Extinction Rates ‘Accelerating’*. IPBES, 2019 (https://www.ipbes.net/news/Media-Release-Global-Assessment).

[4] Steffen W, Rockstrom J, Richardson K, Lenton TM, Folke C, Liverman D, Trajectories of the earth system in the Anthropocene. Proc Natl Acad Sci USA 2018; 115: 8252–59. 30082409 10.1073/pnas.1810141115 PMC6099852

[5] Henrich J, Heine SJ, Norenzayan A. The weirdest people in the world? Behav Brain Sci 2010; 33: 61–83. 20550733 10.1017/S0140525X0999152X

[6] Tol RSJ, Downing TE, Kuik OJ, Smith JB. Distributional aspects of climate change impacts. Glob Environ Change 2004; 14: 259–72.

[7] United Nations International Children's Emergency Fund (UNICEF). The Challenges of Climate Change: Children on the Front Line. UNICEF Office of Research, 2014.

[8] Newnham EA, Titov N, McEvoy P. Preparing mental health systems for climate crisis. Lancet Planet Health 2020; 4(3): E89–90. 32220675 10.1016/S2542-5196(20)30036-X

[9] Sanson AV, Burke SEL. Climate change and children: an issue of intergenerational justice. In Children and Peace: from Research to Action (eds N Balvin, D Christie): 343–62. Springer Publishing 2019.

[10] Sanson AV, Wachs TD, Koller SH, Salmela-Aro K. Young people and climate change: the role of developmental science. In Developmental Science and Sustainable Development Goals for Children and Youth (eds Verma S, Peterson A): 115–38. Springer Publishing, 2018.

[11] United Nations International Children's Emergency Fund (UNICEF). Unless We Act Now. UNICEF, 2015 (https://www.unicef.org/reports/unless-we-act-now-impact-climate-change-children).

[12] Sheffield PE, Landrigan PJ. Global climate change and children's health: threats and strategies for prevention. Environ Health Perspect 2010; 119: 291–8. 20947468 10.1289/ehp.1002233 PMC3059989

[13] United Nations International Children's Emergency Fund (UNICEF) Office of Research. The Challenges of Climate Change: Children on the Frontline. UNICEF Office of Research, 2014 (https://www.unicef-irc.org/e-book/Climate-Ch-web-D215/files/assets/basic-html/index.html#page1).

[14] Garcia D, Sheehan M. Extreme weather-driven disasters and children's health. Int J Health Serv 2016; 46: 79–105. 26721564 10.1177/0020731415625254

[15] Majeed H, Lee J. The impact of climate change on youth depression and mental health. Lancet Planet Health 2017; 1: e94–5. 29851616 10.1016/S2542-5196(17)30045-1

[16] Gibbons E. Climate change, children's rights, and the pursuit of intergenerational climate justice. Health Hum Rights 2014; 16: 19–31. 25474607

[17] Clayton S, Manning C, Krygsman K, Speiser M. Mental Health and Our Changing Climate: Impacts, Implications, and Guidance. American Psychological Association and ecoAmerica, 2017 (https://www.apa.org/news/press/releases/2017/03/mental-health-climate.pdf ).

[18] Burke S, Sanson A, Van Hoorn J. The psychological effects of climate change on children. Curr Psychiatry Rep 2018; 20: 35. 29637319 10.1007/s11920-018-0896-9

[19] Yun K, Lurie N, Hyde PS. Moving mental health into the disaster-preparedness spotlight. New Engl J Med 2010; 363: 1193–4. 20860498 10.1056/NEJMp1008304

[20] Mobjork M. Chapter 8: Exploring the links between climate change and violent conflict. In *SIPRI Yearbook 2017: Armaments, Disarmament and International Security*. Stockholm International Peace Research Institute, 2017.

[21] UN News. *Climate Change Recognized as ‘Threat Multiplier’, UN Security Council Debates its Impact on Peace*. UN News, 2019 (https://news.un.org/en/story/2019/01/1031322).

[22] Fotiadis A. *This Racist Backlash against Refugees Is the Real Crisis in Europe. The Guardian,* 2016 (https://www.theguardian.com/commentisfree/2016/feb/25/racist-backlash-against-refugees-greece-real-crisis-europe).

[23] Pfefferbaum B, Jacobs AK, Van Hoorn RL, Houston JB. Effects of displacement in children exposed to disasters. Curr Psychiatry Rep 2016; 18: 71. 27287465 10.1007/s11920-016-0714-1

[24] Jones A. *The Intersection of Mental Health and Climate Change: Policy Suggestions for Supporting Greenlandic Inuit*. Henry M. Jackson School for International Studies, University of Washington, 2020 (https://jsis.washington.edu/news/the-intersection-of-mental-health-and-climate-change-policy-suggestions-for-supporting-greenlandic-inuit/).

[25] UN General Assembly. *Convention on the Rights of the Child* (Treaty Series No. 1577). UN General Assembly, 1989 (http://www.refworld.org/docid/3ae6b38f0.html).

[26] United Nations International Children's Emergency Fund (UNICEF) UK. *Climate Change: Children's Challenge*. UNICEF UK, 2013 (https://www.unicef.org.uk/publications/climate-change-report-jon-snow-2013/).

[27] Chiw A, Ling HS. *Young People of Australia and Climate Change: Perceptions and Concerns.* Millennium Kids, 2019 (https://www.millenniumkids.com.au/wp-content/uploads/2019/02/Young-People-and-Climate-Change.pdf).

[28] Australian Institute for Disaster Resilience. *Our World Our Say: National Survey of Children and Young People on Climate Change and Disaster Risk.* Australian Institute for Disaster Resilience, 2020 (https://www.aidr.org.au/media/7946/ourworldoursay-youth-survey-report-2020.pdf).

[29] Royal College of Psychiatrists. *The Climate Crisis Is Taking a Toll on the Mental Health of Children and Young People*. Royal College of Psychiatrists, 2020 (https://www.rcpsych.ac.uk/news-and-features/latest-news/detail/2020/11/20/the-climate-crisis-is-taking-a-toll-on-the-mental-health-of-children-and-young-people).

[30] Ojala M. Hope in the face of climate change: associations with environmental engagement and student perceptions of teachers’ emotion communication style and future orientation. J Environ Educ 2015; 46: 133–48.

[31] Strife SJ. Children's environmental concerns: expressing ecophobia. J Environ Educ 2012; 43(1): 37–54.

[32] ReachOut. *New Survey by ReachOut And Student Edge Reveals Number of Students Anxious about Climate Change*. ReachOut, 2019 (https://about.au.reachout.com/blog/new-survey-by-reachout-and-student-edge-reveals-number-of-students-anxious-about-climate-change).

[33] Albrecht G, Sartore GM, Connor L, Higginbotham N, Freeman S, Kelly B, Solastalgia: the distress caused by environmental change. Australas Psychiatry 2007; 5: 595–8. 10.1080/10398560701701288 18027145

[34] Suzuki S. *Speech at U.N. Conference on Environment and Development*. American Rhetoric, 1992 (https://www.americanrhetoric.com/speeches/severnsuzukiunearthsummit.htm).

[35] Rigitano E. *COP24, the Speech by 15-Year-Old Climate Activist Greta Thunberg Everyone Should Listen To*. LifeGate, 2018 (https://www.lifegate.com/greta-thunberg-speech-cop24).

[36] The Guardian. *Climate crisis: 6 million people join latest wave of global protests.* The Guardian, 2019 (https://www.theguardian.com/environment/2019/sep/27/climate-crisis-6-million-people-join-latest-wave-of-worldwide-protests).

[37] National Public Radio (NPR). *Transcript: Greta Thunberg's Speech at the U.N. Climate Action Summit September 23, 2019*. NPR, 2019 (https://www.npr.org/2019/09/23/763452863/transcript-greta-thunbergs-speech-at-the-u-n-climate-action-summit).

[38] Sanson AV, Van Hoorn J, Burke SEL. Responding to the impacts of the climate crisis on children and youth. Child Dev Perspect 2019; 13(4): 201–7.

[39] Hawkins MT, Letcher P, Sanson A, Smart D, Toumbourou JW. Positive development in emerging adulthood. Aust J Psychol 2009; 61: 89–99. 10.1007/s10964-010-9593-7 20936336

[40] Smith EP, Petersen AC, Leman P. Positive youth development in diverse and global contexts. Child Dev 2017; 88: 1035–185. 10.1111/cdev.12860 28598496

[41] Royal College of Psychiatrists. *Eco-distress: For Young People*. Royal College of Psychiatrists, 2020 (https://www.rcpsych.ac.uk/mental-health/parents-and-young-people/young-people/eco-distress---for-young-people).

[42] Australian Psychological Society. A Guide for Parents about the Climate Crisis. Australian Psychological Society, 2018 (https://www.psychology.org.au/for-the-public/Psychology-topics/Climate-change-psychology/Talking-with-children-about-the-environment/A-guide-for-parents-about-the-climate-crisis).

[43] Australian Psychological Society. Raising Children to Thrive in a Climate Changed World. Australian Psychological Society, 2018 (https://www.psychology.org.au/for-the-public/Psychology-topics/Climate-change-psychology/Talking-with-children-about-the-environment/Raising-children-to-thrive-in-a-climate-changed-wo).

